# GPS-DERIVED DAILY MOBILITY AND DAILY WELL-BEING IN COMMUNITY-DWELLING OLDER ADULTS

**DOI:** 10.1093/geroni/igac059.489

**Published:** 2022-12-20

**Authors:** Minxia Luo, Eun-Kyeong Kim, Robert Weibel, Mike Martin, Christina Röcke

**Affiliations:** University of Zurich, Zurich, Zurich, Switzerland; University of zurich, Zurich, Zurich, Switzerland; University of zurich, Zurich, Zurich, Switzerland; University of Zurich, Zurich, Zurich, Switzerland; University of Zurich, Dynamics of Healthy Aging & Center for Gerontology, Zurich, Zurich, Switzerland

## Abstract

Mobility as a multidimensional concept has rarely been examined as a day-to-day varying phenomenon in its within-person association with older adults’ daily well-being. Using a custom-built mobile GPS sensor („uTrail“) combined with a smartphone-based ambulatory assessment, this study examined associations between daily mobility and daily well-being in community-dwelling older adults. Analysis included 947 days’ data from 109 Swiss older adults aged 65 to 89 years. Multilevel modelling showed that, within persons, a day with larger life space area, more time spent in passive transport modes, and higher number of different locations was associated with higher daily life satisfaction, but not daily positive or negative affect. Follow-up analysis showed that daily maximum distance from home was positively associated with daily life satisfaction, providing a first indication that exposure to non-habitual environments might be a candidate mechanism to explain effects of mobility. Results are discussed in the context of healthy aging research.

